# A Warm Welcome to The First Issue of *JSCAI*

**DOI:** 10.1016/j.jscai.2021.100013

**Published:** 2022-01-30

**Authors:** Alexandra Lansky

It is my great privilege to have been entrusted with the launch of the *Journal of the Society for Cardiovascular Angiography and Interventions* (*JSCAI*). *JSCAI* is a powerful vehicle for authors to contribute and disseminate new clinical and scientific evidence related to all aspects of interventional cardiovascular medicine. As we release our first issue of the *JSCAI*, I hope you will enjoy the content and be motivated to contribute to our interventional community by submitting your next scientific manuscript.

Our first issue of *JSCAI* leads off with the SCAI SHOCK Stages Classification Expert Consensus Update chaired by Srihari S. Naidu, MD.^1^ The SCAI SHOCK Stage Classification System was created to be a simple, broadly applicable tool for clinicians using limited clinical information. The expert consensus update incorporates validation studies confirming the discriminatory risk of the model, with a consistent rise in mortality with each increasing SCAI SHOCK stage. Further refinement of the classification proposes a “3-axis model” that integrates the SCAI SHOCK stages, patient variables, and “shock phenotypes” (acute myocardial infarction, acute on chronic heart failure, isolated right ventricular failure), providing a prognostically relevant matrix that can be readily incorporated in practice. This framework is foundational for future efforts to establish best treatment practices including pathways of care, best treatment options, and timing for each stage of shock. This consensus document will be a valuable reference and is widely relevant to all interventional practices.

Our first *JSCAI* issue features 2 original manuscripts on the treatment of severely calcified coronary lesions using intravascular lithotripsy (IVL). Interventionalists are confronted with severely calcified coronary lesions in almost one-third of coronary interventions, and these remain among the more challenging lesion types, with high acute procedural complications and worse long-term outcomes. Dean J. Kereiakes, MD, and colleagues report the 1-year results of the pivotal Disrupt CAD III study.^2^ IVL delivers acoustic pressure waves to modify calcium, enhance vessel compliance, and optimize stent deployment. In Disrupt CAD III, IVL treatment prior to coronary stent implantation in severely calcified lesions was associated with low rates of 1-year major adverse cardiac events, ischemia-driven target lesions revascularization, and stent thrombosis, extending the benefits previously observed with IVL at 30 ​days. This study will change practice and is a must read for all coronary interventionalists.

Yasin Hussain, MD, and colleagues report sex-based outcomes after coronary IVL in a large patient-level pooled analysis of the 4 prospective Disrupt CAD studies.^3^ Women undergoing percutaneous coronary intervention of severely calcified lesions are particularly vulnerable to poor acute and long-term outcomes, including more flow-limiting dissections, perforations, and a higher rate of mortality in the longer term compared with men. The evidence presented in the current issue with IVL-facilitated stenting of severely calcified lesions demonstrates low rates of dissection and perforation and similar safety and efficacy in women and men. IVL may well become the preferred interventional therapy for plaque modification of severely calcified lesions in women.

Kush Patel, MD, and colleagues report the results of a new biodegradable polymer sirolimus-eluting stent from the large, randomized PIONEER III trial in complex coronary lesions.^4^ The new Supreme DES targets early drug elution and polymer degradation within 4 to 6 ​weeks, leaving behind a stent surface with an electrografted, nanometric, biostable coating that promotes healing. Compared with the durable polymer Xience stent, the Supreme DES had a similar rate of target lesion revascularization at 12 ​months in complex lesions, defined as Type B2/C based on the American College of Cardiology/American Heart Association criteria. Patients will be followed up for 5 ​years to establish whether there is long-term benefit with the early drug elution and polymer degradation of the Supreme DES.

Tayyab Shah, MD, examined the impact of sex and timing on mechanical circulatory support (10.13039/100011715MCS) in myocardial infarction complicated by cardiogenic shock.^5^ This study offers a glimpse into potential disparities in advanced therapies in women compared with men with acute myocardial infarction with cardiogenic shock. Women had more escalation of inotropes prior to MCS, more intra-aortic balloon pump use prior to escalation, delays in initiating MCS, and shorter duration of MCS. The authors suggest that early initiation of MCS rather than escalation of inotropes may improve outcomes in women.

In a research letter, Gilbert H.L. Tang, MD, discusses strategies including IVL and balloon dilation of expandable sheaths to facilitate transfemoral SAPIEN 3 transcatheter aortic valve replacement in severely calcified vessels; Dhananjai Menzies, MD, describes a no-contrast conscious sedation approach for patients at high risk for acute kidney injury; and Kashish Goel, MD, presents a complex left main bifurcation case treated with orbital atherectomy using microcatheter protection.

This compilation is accompanied by 2 editorials by Dr Sandeep Nathan (SCAI SHOCK consensus)^6^ and by Dr Antonio Colombo (Disrupt CAD III),^7^ and a series of recorded roundtable discussions from leading experts in the field who will discuss the findings and contextualize the results and implications for your daily practice. I hope you will find these informative and enjoyable.

## Our editorial team

I am grateful to our outstanding deputy and associate editors, whose enthusiasm, collaborative spirit, and commitment to deliver high scientific content has been unwavering. I want to thank our growing editorial board that now exceeds 200 members and represents a diverse, highly talented, international interventional community that supports our educational goals through outstanding reviews and feedback in record time. My gratitude to the Elsevier editorial and production teams (Jane Grochowski, Colin Conway, and David Newcombe) and the SCAI publication group (Robert Bartel, Amanda Pettyjohn, and Chris Trimmer), who have been invaluable in the launch of the *JSCAI*. Lastly, my sincere gratitude to our Executive Editor, Dominic Francese, whose dedication and professionalism have made this launch enjoyable and almost seamless!

Join us in making *JSCAI* a top-ranking impactful journal to advance interventional practice and patient outcomes. Submit your science to *JSCAI*!
